# Cholera Inoculation

**Published:** 1892-09-17

**Authors:** 


### The Hospital

An Institutional Journal of

Science, Medicine, Nursing, and Philanthropy.

*For Hospitals, Asylums, Infirmaries, Dispensaries, Medical Practitioners, Students, Nurses, and the Charitable Public.*

Vol. XII.—No. 312.] [Sept. 17, 1892.

### Cholera Inoculation.

Mr. E. H. Hankin, one of the best known of our younger bacteriologists, has had himself inoculated with the cholera bacillus. M. Haffkine, of the Pasteur Institute, was the operator. M. Haffkine himself and seven or eight other enthusiastic scientists have all undergone the cholera inoculation. These gentlemen have to a limited extent shown the courage of their convictions; each of them has experienced certain novel sensations; they are all at this moment alive and well; but are they protected against cholera? This is, for them and their methods, and for a deeply-interested generation, the question of questions.

The story of Mr. Hankin's experience is peculiarly interesting, not only at the present moment, but permanently. Moreover, like all "the fairy tales" of *bona fide* "science," it has the merit of being "true in substance and in fact," as the lawyers say. When this scientific enthusiast went to Paris he found that M. Haffkine had developed a system of inoculation of considerable complexity. The original intention in cholera inoculation had been to use a virus of somewhat intensified potency. But the use of such a virus had resulted in certain practical inconveniences, the chief of which was the death and subsequent separation of a considerable mass of tissue at the place of inoculation. It was therefore felt to be desirable that some preliminary steps should be taken which would modify the severity of the local effects of the strong virus whilst allowing the use of the virus itself for the sake of its more efficient protecting power. The method of inoculation which was finally decided upon consisted of two parts—a preliminary inoculation with a true cholera virus artificially attenuated, and a final inoculation, eight or ten days later, with a true cholera virus artificially intensified. This double-barrelled inoculation was what Mr. Hankin submitted himself to, and his experiences connected therewith constitute his most interesting report to the *British Medical Journal*.

Mr. Hankin was first inoculated on August 16th with the attenuated virus. In about an hour he began to feel a little pain at the point of inoculation; in four hours the pain had increased considerably, and the part had begun to swell; in eight hours the pain and swelling were so decided that it was distressing to move about; in ten hours the temperature had begun to rise; in twelve hours Mr. Hankin felt ill, and the temperature had reached 100 deg. Fahrenheit. At this point the unpleasant symptoms seem to have reached their maximum intensity. Next morning the patient waked up and found himself feeling better, and that his temperature had become normal. From this time the pain and swelling gradually passed away, and in the course of four or five days most of the symptoms had disappeared. This was the preliminary and preparatory inoculation; and physiologists will see at once that the amount of disturbance produced in the system was quite unimportant.

On August 21st, five days after the first inoculation, Mr. Hankin felt himself ready to undergo the second and essential operation, the inoculation with the intensified virus, which was to render him quite impervious to future attacks of cholera. The following were, in brief, the symptoms in their ordered succession: The inoculation was made at six o'clock in the evening, about the centre of the inner side of the left arm; in seven hours the arm was painful all the way up to the armpit, and there was slight intermitting headache; five hours later, that is, about four o'clock in the morning, Mr. Hankin waked up, and found it difficult to get out of bed on account of the pain; in a few minutes, however, getting accustomed to change of position, he felt able to walk about with ease, but he was still ill and feverish; at nine o'clock he ate his breakfast with a good appetite. During the greater part of the day after the operation he slept, only being awakened to have his temperature taken; in the evening he felt bilious and constipated; the arm was swollen, red, and painful. On the third morning he got up feeling quite well; the pain had diminished, but the swelling had increased, though the skin was no longer red. On the fourth day after the operation the swelling had still further diminished, but the area of reddened skin had turned yellow. In another day or two most of the symptoms had disappeared. The net result of each operation, which is visible to the eye, is a small, hard nodule, less than half an inch in diameter, at each seat of injection.

It has seemed desirable to give an account of the two operations in a little detail, in order that readers may understand the degree of severity of the constitutional symptoms set up by this method of prophylaxy; for it is obviously undesirable to give encouragement to measures of prevention which may themselves prove to be more dangerous than the disease to be prevented. The operation submitted to by Mr. Hankin has been practised upon eight or nine other persons, and none of them have had more severe constitutional symptoms than he experienced: most of them, indeed, suffered less.

But now comes the real question at issue: Are Mr. Hankin and his companions, or are they not, protected against cholera invasion? The answer is, that they do not appear as yet to have exposed themselves to any risks. They seem to think that they are reasonably safe; but they surely do not need to be reminded that experiments which work out successfully in the laboratory do not invariably work out successfully in the unscientific, every-day world. The agent employed in the inoculation was after all only a "cultivation" bacillus. M. Haffkine's own experiments with guinea pigs on this very subject show how exceedingly easy it is to modify the character of a virus by cultivation and other methods. It is as plain as possible that laboratory experiments are in no sense conclusive; they point the way, and that is all. We are not forgetting the results of the various inoculations as seen in guinea pigs. We accept with absolute confidence the statement that some of the guinea pigs inoculated with the intensified virus were quite immune against the cholera bacillus in whatever way it was introduced into the system. But even in the case of the guinea pigs Mr. Hankin leaves us in doubt whether they were immune against natural cholera dejecta, or only against the cultivations of the laboratory. This is a point on which we should have been furnished with the fullest and clearest information.

There is one experiment which suggests itself to practical minds: it is that M. Haffkine and his friends should betake themselves to Hamburg, and there set to work with natural cholera dejecta on the very spot where all the conditions of a normal epidemic are in full and energetic action. The guinea pigs that are believed to be immune should be submitted to inoculations and other methods of administration of natural cholera dejecta in every form. If the guinea pigs should prove themselves to be actually immune under these circumstances, then one more experiment should obviously follow. M. Haffkine, Mr. Hankin, and as many other enthusiastic disciples of inoculation as can be got together, should submit themselves to the test of natural cholera dejecta, administered in sufficient quantities, moist and dry, *per os*, *per rectum*, by inoculation, and in every other way which can suggest itself to ingenious laboratory physiologists. This, we submit, is not too extreme a demand. It is simply asking the experimenters to have the full courage of their convictions, and to put their work to the only proof which is absolute and incontestable. If such a test as this were undertaken, and were to be justified by a successful issue, then indeed a most notable advance in prophylactic medicine would have been made and demonstrated.

